# São Paulo Medical Journal/ Revista Paulista de Medicina on the Scientific Electronic Library Online (SciELO)

**DOI:** 10.1590/S1516-31802000000200001

**Published:** 2000-03-02

Since 24 October 1999, *São Paulo Medical Journal/ Revista Paulista de Medicina* has been on the internet as a part of the SciELO (The Scientific Electronic Library Online). The reader will find current and previous issues (since 1998); the past issues will be added on a regular basis. SciELO is an electronic virtual library covering a selected collection of Brazilian scientific journals. The library is an integral part of a project being developed by FAPESP - Fundação de Amparo à Pesquisa do Estado de São Paulo, in partnership with BIREME - the Latin American and Caribbean Center on Health Sciences Information.

The FAPESP-BIREME Project envisages the development of a common methodology for the preparation, storage, dissemination and evaluation of scientific literature in electronic format.^1^ As the project develops, new journal titles will be added to the library collection. The objective of the site (www.scielo.br) is to implement an electronic virtual library, providing full access to a collection of serial titles, a collection of issues from individual serial titles, as well as access to the full texts of articles. Access to both serial titles and articles is available via indexes and search forms. The site will be constantly updated both in form and content, as the project advances.

SciELO's interface provides access to its serial collection via an alphabetic list of titles or a subject index or a word search for serial titles, publisher names, city of publication and subject. The interface also provides access to the full text of articles via author index or subject index or a search form on article elements such as author names, words from title, subjects and words from the full text.

The following advantages and benefits to the publishing and distribution processes for scientific documents are described in SciELO Project^1,2^:

- automatic publication, distribution and indexing of scientific texts
- increase and acceleration in the capacity for scientific communication
- standardization of the structure of electronic publications, avoiding the pulverization of formats, methods and location
- automatic bibliographical control and standardized access to data
- systematic and consistent evaluation of the scientific literature
- economy in the training in, use of and dissemination of methodology
- cost-cutting and rationalization in the production of electronic publications
- standardization and flexibility for electronic publication databases
- added value for the producer (researchers, scientists, editors and publishers), for the user (researchers, scientists and students) and for the information manager (librarians and document handlers)
- increase in the visibility of scientific research

The *São Paulo Medical Journal / Revista Paulista de Medicina* and a selected collection of Brazilian scientific journals are available on a single website with the same access interface (www.scielo.br). Just like the Electronic British Medical Journal (www.bmj.com), it is full text, with free access, and we do not ask you to pay, subscribe, register, provide a password, or fill in interminable online questionnaires.

Created as *Revista da Associação Paulista de Medicina* in 1932, changed to *Revista Paulista de Medicina* in 1942, and transformed into full text in English in 1992, we are just at the start of a new stage of the journal, and thus both electronic and paper publication will be maintained. However, a time gap is expected to appear between the electronic and printed versions. The electronic version will become, by far, the first one available to the reader, and probably the more useful.

Papers published in SPMJ have been indexed in MedLine since 1965. A search done by journal name showed 1,949 records (PubMed 17Dec1999). And furthermore, there is a link to go to the publisher site (SciELO site): if anyone wants to search in PubMed, it is possible to access the full text of SciELO journals, thereby increasing the accessibility of Brazilian journals.
